# State of emergency contraception in the U.S., 2018

**DOI:** 10.1186/s40834-018-0067-8

**Published:** 2018-09-05

**Authors:** Kristin O. Haeger, Jacqueline Lamme, Kelly Cleland

**Affiliations:** 1 Department of Veterans Affairs, Veterans Health Administration, Office of Patient Care Services, Women’s Health Services, 810 Vermont Ave., NW, Washington, DC, 20420 USA; 2 Department of Obstetrics & Gynecology, U.S. Naval Hospital Okinawa, Okinawa, Japan; 3 Office of Population Research, Princeton University, 218 Wallace Hall, Princeton, NJ 08544 USA

**Keywords:** Emergency contraception, Levonorgestrel, Plan B One-Step, Ulipristal acetate, Ella, Copper IUD

## Abstract

Emergency contraception is indicated in instances of unprotected sexual intercourse, including reproductive coercion, sexual assault, and contraceptive failure. It plays a role in averting unintended pregnancies due to inconsistent use or non-use of contraception. Options for emergency contraception vary by efficacy as well as accessibility within the U.S. This paper provides an overview of levonorgestrel (Plan B One-Step and generic counterparts), ulipristal acetate (sold as ella), and the copper intrauterine device (IUD, sold as ParaGard), including the mechanisms of action, administration, efficacy, drug interactions, safety, side effects, advantages, and drawbacks. It will also review current misconceptions about emergency contraception and access for subpopulations, including adolescents, immigrants, survivors of sexual assault, rural populations, and military/veteran women. This paper will address barriers such as gaps in knowledge, and financial, health systems, and practice barriers. Continuing areas of research, including the impact of body weight on the efficacy of emergency contraceptive pills and potential interactions between ulipristal acetate and ongoing hormonal contraceptives, are also addressed.

## Background

In the US, 45% of pregnancies were unintended in 2011, dropping from 51% in 2008 [1]. Although 68% of women at risk for unintended pregnancy consistently used contraception, these women account for only 5% of unintended pregnancies [2]. The vast majority of unintended pregnancy is due to inconsistent use or non-use of contraception, and this is where emergency contraception has a potentially important role [2]. Indications for emergency contraception include any situation in which sexual intercourse is unprotected, including reproductive coercion, sexual assault, and contraceptive failure.

**Fig. 1:**
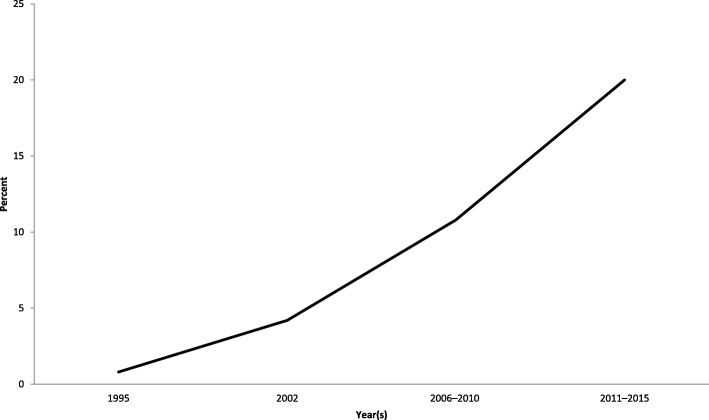
Ever-use of emergency contraception among women aged 15–44 who have ever had sexual intercourse. Based on National Center for Health Statistics using National Survey of Family Growth, 2013 and 2017 [3, 104]

Since 1995, ever-use of emergency contraception has increased among women age 15–44 from 0.8% in 1995 to 20.0% from 2011 to 2015 (see Fig. 1) [3]. Multiple options for emergency contraception exist; however, the options vary in efficacy. Among both medical providers and the general population, there is a gap in knowledge as well as persistent misperceptions about the options for emergency contraception, the timing, side effects, and mechanism of action [4–10]. Our intent is to give an overview of the options for emergency contraception currently available in the United States; address misconceptions about emergency contraception and issues of access and barriers; review emergency contraception in special populations; and touch on future research.

## Levonorgestrel

Levonorgestrel (LNG) is a progestin-only emergency contraception pill (ECP) that should be taken orally as soon as possible, within a 72-h window following sexual intercourse, although some studies suggest moderate efficacy up to 120 h post-coitus [11–14]. It is sold in the United States as Plan B One-Step® (1.5 mg) as a single dose and is sold under several generic labels, including Take Action, My Way, AfterPill (available only online), Aftera, and Option 2. It sometimes referred to colloquially as “the morning-after pill” and was originally sold as Plan B, which included two doses (0.75 mg) to be administered 12 h apart before studies showed the single dose to be as effective as the staggered doses. The two-dose version is no longer sold in the United States.

### Mechanism of action

The primary mechanism of action of LNG is suppression of luteinizing hormone, which delays or inhibits ovulation [15–22]. Studies are inconclusive in showing whether it may also interfere with fertilization by thickening cervical mucus and impairing tubal transport of sperm or the egg [19, 22–27]. The best available evidence suggests that progestin-only ECPs work only before ovulation has occurred and do not inhibit implantation of a fertilized egg [15, 28]. Progestin-only ECPs are ineffective after an embryo has implanted in the uterus and thus cannot work as an abortifacient [15, 29].

### Administration: Timing and importance of long-term contraception plans

No physical exams, laboratory tests, or pregnancy tests are required before taking LNG [30]. It can be administered regardless of timing of a patient’s menstrual cycle [14]. When post-coital emergency contraception is needed, when feasible and welcome, it is recommended that providers offer counseling on long**-**term contraception plans for patients as well as physical and psychological assessments in instances of sexual assault/rape, while being sensitive to women who are not emotionally ready to talk [14, 30]. In the case that the patient vomits within 3 h of consumption of LNG, the patient should take a second dose [13, 14]. Subsequent to taking LNG, it is safe to start or resume ongoing contraception methods immediately, sometimes referred to as quick-starting [13, 14].

### Efficacy

Plan B One-Step (1.5 mg) has a half-life of approximately 27.5 ± 5.6 h [31]. A series of randomized trials examining the efficacy of LNG have demonstrated that for the single-dose regimen (1.5 mg), the failure rate ranges from 0.3–2.6% [11, 32, 33].

LNG may be used after a single episode of unprotected intercourse (UPI) [14]. If a patient has UPI again after taking LNG, she will need to use a backup method of contraception because LNG does not prevent against future acts of intercourse (although it is not necessary to take it more than once within 24 h) [14]. It is safe to use a second dose of LNG within the same menstrual cycle for a subsequent episode of UPI [14, 30]. A study of the use of 1.5 mg of LNG as a primary method of contraception found that the method was about as effective as other coitus-dependent methods, and was highly acceptable to women. Side effects were generally mild and transient, although some women experienced changes in menstrual patterns [34].

Recently, studies have investigated the attenuation of the efficacy of LNG for post-coital emergency contraception across weight and body mass index (BMI). Evidence suggests that the efficacy of 1.5 mg of LNG may decrease among patients weighing more than 75 kg or with a BMI greater than 26 kg/m^2^ [35–37]. Recent pharmacokinetic studies of 1.5 mg LNG show that the blood serum level in women with an obese BMI (greater than or equal to 30) was 50% that of women with a normal BMI (between 18.5 and 25 kg/m^2^), and that doubling the dose to 3.0 mg in women with an obese BMI resulted in equal serum concentrations of LNG as the 1.5 mg dose in normal-BMI women [38]. Therefore, if other EC options are not available, providing a double dose of LNG may be a reasonable recommendation for women with an obese BMI [38, 39].

### Drug interactions

Drugs that may reduce LNG plasma levels include barbiturates, bosentan, carbamazepine, felbamate, griseofulvin, oxacarbazepine, phenytoin, rifampin, St. John’s wort, topiramate, and certain anti-retroviral therapies [14, 40]. Because the efficacy of LNG EC for women using enzyme-inducing drugs may be compromised, a double dose of LNG (3.0 mg) is recommended, although the effectiveness of this approach is unproven [14].

### Safety/contraindications

There are no risks of LNG EC that outweigh the benefits of preventing an unintended pregnancy and no deaths attributed to its use [30, 40, 41]. Contraindications in instances of ongoing levonorgestrel-containing contraception (e.g., cardiovascular disease, migraines, liver diseases, breastfeeding, and thromboembolic complications) are mediated for EC users by the short-term exposure and relatively low quantity of hormones consumed [14]. Therefore, women with these contraindications to hormonal birth control use may still use LNG EC [40]. Known or suspected pregnancy is the only contraindication for LNG EC; this is not because LNG EC would harm an existing pregnancy, but because it will not be effective. Studies show that it has no documented teratogenic effects on fetuses or subsequent indications of birth defects [30, 40]. For patients who use LNG EC and experience a treatment failure, there is no heightened risk of ectopic pregnancy [42]. Breastfeeding is not a contraindication to using LNG EC [14, 40].

### Side effects

Documented side effects include the following: nausea (13–23%), vomiting (5.6%), abdominal pain (13–18%), fatigue (13–17%), dizziness (9–11%), headaches, and breast tenderness [11, 32, 43]. Most symptoms subside within 24 h of administration of the pill [29]. In instances when LNG is taken in the preovulatory stage of menses, the length of the cycle may be abbreviated; in the peri- and postovulatory phases, cycle length is unaffected, but the duration of bleeding is elongated in the subsequent cycle [44–46].

### Pros/cons

The potential ease of access of LNG EC is beneficial for many patients. Within the U.S., LNG EC 1.5 mg (Plan B One-Step and generics including Take Action, My Way, and Aftera) is available over the counter without restrictions by age or gender; additionally, no identification is required for purchase [7]. Typically it costs $50 for the brand name and $40 for a generic version. Individuals can purchase lower-cost LNG EC online for $20 plus $5 shipping online for advanced provision. However, LNG EC is less effective than other EC options (ulipristal acetate or the copper IUD), particularly for women who are obese or overweight [14].

## Ulipristal acetate

Ulipristal acetate (UPA) is an antiprogestin sold in the U.S. as ella® (30 mg). It should be taken as soon as possible post-coitus and remains effective for 120 h following intercourse [14, 40].

### Mechanisms of action

Like LNG, ulipristal acetate delays ovulation [14]. It does not appear to impair sperm function [47]. Some studies have shown that UPA may alter certain endometrial parameters, although in-vitro studies show no difference in the ability of embryos to implant when exposed or not exposed to UPA [48]. The best available evidence suggests that the primary mechanism of UPA in doses used for EC is interference with ovulation [16, 49]. Because UPA works primarily by delaying ovulation, it may only be used for a single episode of UPI. For subsequent episodes of intercourse within that menstrual cycle, additional contraception methods will be required to prevent an unintended pregnancy.

### Administration: Timing and importance of long-term planning

Since both UPA and progestin-based contraceptives bind to progesterone receptors, patients are advised to wait for at least 5 days between the administration of UPA and starting/resuming a hormonal contraceptive [13, 14]. The packaging on ella further recommends the use of barrier protection (e.g., a condom) until the patient’s next menstrual cycle.

One study evaluated repeated use of ulipristal acetate within the same cycle; women took UPA either once every 5 days or once every 7 days for 8 weeks [50]. No safety concerns emerged, although most women ovulated at some point during the study. This suggests that while repeated use of UPA within the same cycle is safe, it may not be suitable for use as an ongoing, regular method because users are likely to ovulate at some point and therefore be at risk for pregnancy [50].

### Efficacy

Overall, ulipristal acetate is more effective than LNG in the first 72 h post-coitus and is the only US Food and Drug Administration (FDA)-approved ECP for use between 72 and 120 h post-coitus with a half-life of 32.4 ± 6.3 h [11, 51]. Randomized trials evaluating the efficacy of UPA (30 mg) have documented failure rates ranging from 0.0–1.8% with no decrease in efficacy over the 120 h period unlike LNG, which does decrease in efficacy over that timeframe [11, 43, 49]. A meta-analysis of two randomized trials directly found that UPA to be more effective than LNG; the odds of pregnancy for UPA compared with LNG were 42% lower up to 72 h and 65% lower in the first 24 h [11].

A randomized pharmacokinetic study compared LNG and UPA in women with a normal BMI in the control group with those with an obese BMI [38]. Relative to control subjects, those with a BMI greater than 30 using UPA had similar serum levels, while those using LNG had serum levels about half of the control group [38].

### Drug interactions

Hepatic enzyme-inducing medication ingested in the previous month may reduce the efficacy of ulipristal acetate [14, 40]. Furthermore, drugs intended to increase levels of gastric pH, including antacids, histamine H2 antagonists, and proton pump inhibitors, are also contraindicated [14, 40]. Doubling the dose of UPA in women using these medications has not been studied and is not recommended at this time [14]. Because UPA is an antiprogestin, it is likely to compete with ongoing progestin-based contraceptive methods, which is why it is recommended that patients wait 5 days prior to starting a hormonal method of contraception after UPA use [14].

### Safety

As with LNG, there are no risks of UPA that outweigh the benefits of preventing an unintended pregnancy and no deaths have been attributed to it [13]. It is well-tolerated in studies evaluating its repeated use over time [41, 50], although the FDA label for ella currently indicates that is not intended for this purpose [52].

No teratogenic effects or birth defects have been associated with UPA taken during pregnancy in the case it is inadvertently taken by a woman who does not realize she is already pregnant [14, 40]. Though there are no adverse outcomes associated with breastfeeding after taking UPA [40], the ella label discourages it. U.S. guidelines recommend mothers avoid giving breastmilk in the 24 h following consumption of UPA [40], while European guidelines suggest a 7-day window before resuming breastfeeding an infant following the ingestion of UPA [14].

### Side effects

Side effects of ulipristal acetate include delayed menses by 2.1 days (standard deviation ± 8.2 days), headache (19%), dysmenorrhea (13%), nausea (13%), fatigue (6%), dizziness (5%), abdominal pain (5%), upper abdominal pain (3%), and back pain (3%) [11, 49].

### Pros/cons

The primary advantage of ulipristal acetate is that it has greater efficacy than LNG and is the only ECP labeled for use 72–120 h after UPI [11, 13, 30]. Additionally, it is more efficacious for overweight and obese women than LNG [38]. However, unlike the LNG ECP at this time, UPA is only available by prescription, and it is not immediately available in many pharmacies in the United States [53]. In some states, pharmacists are authorized to directly prescribe ulipristal, but in most states, a patient must get a prescription from a provider [54]. UPA can be purchased through online pharmacies for $67, including consultation with a physician and shipping.

## Copper IUD

The most effective form of emergency contraception is the copper intrauterine device (IUD) (see Table 1) [16, 40]. Most guidelines recommend insertion within 5 days of ovulation following an episode of UPI, although one study showed that the copper IUD is effective for emergency contraception at any point in the menstrual cycle, as long as pregnancy has been ruled out [55]. For the sake of parsimony and clarity, World Health Organization (WHO) guidelines recommend insertion within 5 day post-coitus [56]. In the U.S., it is sold as ParaGard® T 380A Intrauterine Copper Contraceptive.

**Table 1:** Comparison of methods of emergency contraception

	Copper IUD	UPA	LNG
Efficacy^a, b, c, d^	1 Most effective overall	2 Not as effective as copper IUD; most effective ECP	2 Less effective than copper IUD or UPA
Timing of use^b, c, d, e, f^	Typically 5 days after UPI (120 h), but may be effective at any point in the cycle	5 days after UPI (120 h)	3 days after UPI (72 h), although may have efficacy up to 120 h
Available OTC^c, g^	No	No	Yes
Timing of long-term birth control after use^b, c^	Leave in for continued use for up to 12 years	Wait 5 days	Immediately (quick-start)
Dosage^b, g^	N/A, insertion by medical provider	30 mg, one dose	1.5 mg, one dose
BMI^g, h^	1 No decrease in efficacy by BMI	2 Decrease in efficacy for BMI ≥ 30	2 Decrease in efficacy for BMI ≥ 25
Breastfeeding^e^	1	1	1
Hx of severe cardiovascular disease^e^	1	2	2
Migraine^e^	1	1	1
Severe liver disease^e^	1	2	2
CYP3A4 inducers^e^	1	2	2

*IUD* intrauterine device, *UPA* ulipristal acetate, *LNG* levonorgestrel, *ECP* emergency contraception pill, *UPI* unprotected intercourse, *OTC* over-the-counter, *N/A* not applicable, *mg* milligrams, *BMI* body mass index, *Hx* history. CDC MEC Categories for classifying emergency contraception: “1 = A condition for which there is no restriction for the use of the contraceptive method; 2 = A condition for which the advantages of using the method generally outweigh the theoretical or proven risks” ^b^

^a^ACOG Committee Opinion [68],

^b^Curtis, Jatlaoui, Tepper, et al. [13],

^c^Trussel, Raymond, Cleland [29],

^d^FSRH, 2017 [14]

^e^Curtis, Tepper, Jatlaoui, et al. [40],

^f^Emergency contraception should be taken as soon as possible following UPI

^g^ACOG Practice Bulletin [30],

^h^Glasier, Cameron, Blithe, et al. [35],

### Mechanism of action

The T-shaped device is wrapped in copper coil that releases copper ions. While the mechanisms of action are not fully understood [57], it is known that the copper ions reduce sperm motility in passing through cervical mucus and create a hostile environment for the sperm [57]. Copper can also alter the uterine and tubal environment and increase prostaglandins, enzymes, and white blood cells, although this is not believed to disrupt an already-implanted blastocyst [16].

### Administration

A clinician should first reasonably rule out pregnancy and assess for medical contraindications, including active infection with gonorrhea or chlamydia, which may put the patient at risk for a pelvic inflammatory disease [13]. A copper IUD may be inserted into the uterus by a trained medical provider at any point during a menstrual cycle as long as pregnancy has been ruled out [40]. Upon insertion of the IUD, the patient may resume all activities, including sex and exercise, as soon as the patient feels ready.

Upon insertion, the IUD will prevent a pregnancy for that episode of UPI as well as subsequent episodes for at least 12 years as long as the IUD remains in place, and no other method of birth control is needed [29]. The provider should still discuss risks of sexually transmitted infections, including HIV, which copper IUDs do not protect against [13]. If menses does not start on time following insertion of the copper IUD, a urine pregnancy test is recommended [14].

### Efficacy

The copper IUD is the most effective form of emergency contraception available [13, 14, 30]. Failure rates are less than 0.1% with only 10 pregnancies documented for over 7000 post-coital insertions [29, 58]. Copper IUDs do not vary in efficacy by BMI, and are therefore the best option for patients without other contraindications with a BMI above 25 [14, 40].

### Drug interactions

There are no known drug interactions [14].

### Safety/contraindications

Contraindications to the copper IUD placement for emergency contraception are the same as at any other time of IUD placement. Contraindications in which the risks outweigh the benefits include pregnancy; untreated cancer of the uterus, cervix, or genital tract; unexplained vaginal bleeding; malignant gestational trophoblastic disease; current pelvic inflammatory disease; Wilson’s disease; uterine malformation preventing insertion; pelvic tuberculosis; a copper allergy; or active gonorrhea or chlamydia [56]. WHO guidelines indicate the following conditions are contraindications to use unless an unsuitable alternative is found, in which case it may be considered under close medical supervision: benign gestational trophoblastic disease; ovarian cancer; HIV/AIDS which is not virally suppressed; or being 48 h to 4 weeks postpartum, when risk of IUD expulsion and uterine perforation is heightened [56]. Relative to women not using any form of contraception, the absolute risk of ectopic pregnancy is lower among women using copper IUDs, with 3.0–4.5 ectopic pregnancies per 1000 person-years without contraception versus 0.2 with a copper IUD [57]. The risks of insertion of the copper IUD for EC are the same as they are for placement of the copper IUD in a patient switching from another contraceptive method. There is no evidence of increased birth defects due to use of a copper IUD [40]. There are no contraindications for breastfeeding with a copper IUD as there are no hormones present to alter breastmilk [40].

### Side effects

Insertion of copper IUDs is associated with increased risk of menstrual cramping, heavier periods, irregular menses, anemia, back pain, and fainting immediately following insertion [14]. Some patients may also experience discomfort at the time of insertion.

### Pros/cons

Pros of copper IUDs are the long-term efficacy for the subsequent 12 years following an episode of UPI as well as consistent efficacy regardless of the patient’s BMI [40]. A limitation to access is that copper IUDs must be inserted by a trained medical provider. Patients in the U.S. who have coverage through a private provider or the Affordable Care Act (ACA) may be able to acquire a copper IUD for little or no cost as long as the ACA remains in effect [6, 58]. Otherwise, the cost may be prohibitively expensive, costing up to $1300 out-of-pocket in the U.S. including both the device and the cost of insertion [59]; however, if the IUD is kept in place, over the course of the following decade it is highly cost-effective [14, 29]. An IUD still costs less than carrying a pregnancy to term for most women when collateral costs, healthcare expenses, leave from work for prenatal checkups, expenses related to giving birth, and other incidentals are factored in. A further limitation is lack of awareness on the part of both patients and providers about the utility of copper IUDs as a form of emergency contraception [60]. Additionally, some healthcare providers adhere to unnecessary protocols requiring two visits for insertion, adding dual burdens of cost and inconvenience for the patient [61].

## Other options

Although not marketed specifically for emergency contraception, a precursor to current methods was a combination of oral contraceptive pills, referred to as the “Yuzpe regimen.” Historically, women would combine pills containing ethinyl estradiol (100 mcg) and LNG (0.50 mg) with a second dose 12 h later. This method lacks the efficacy of more modern interventions [56], has more severe side effects, and is no longer recommended unless other options for EC are not available.

Mifepristone, previously known as RU-486, is an antiprogestin that is highly effective as emergency contraception, with an excellent safety and side-effect profile [62]. It is sold as EC in doses of 10 to 25 mg in several countries, including Armenia, Moldova, Ukraine, China, Russia, and Vietnam [29]. Mifepristone is also used, at much higher doses, to induce abortion.

Currently, studies of LNG IUD alone as a form of emergency contraception are underway [14]. A small prospective cohort study among 188 EC users examining simultaneous use of a LNG IUD and oral LNG EC pills showed that women were twice as likely to choose a combination of oral LNG (1.5 mg) and a LNG IUD compared to a copper IUD [63]; with the exception of one woman who had multiple episodes of UPI, there were no pregnancies in either group. Alternative forms of EC are currently under investigation. However, little data is available on their efficacy at this time.

## Misconceptions about EC

Myths and misconceptions persist regarding emergency contraception that may interfere with its use, including perceptions that emergency contraception acts as an abortifacient [64–66]. Another persistent myth is that patients on other forms of birth control would have no reason to take emergency contraception or that patients would not be able to follow the directions to take an advanced provision of EC [67, 68]. There are many reasons why a person using another form of birth control could require EC, for example, if a condom breaks, a patient forgets to consistently take ongoing hormonal contraceptives, or an IUD is expelled. Women, and even some providers, believe that because they are nulliparous, they are not able to get an IUD [69]. Finally, there is an unfounded belief that the availability of EC could lead to increased risk-taking behavior among teens [70–72]. One of the biggest hurdles to increasing uptake of EC is dispelling myths and increasing knowledge among providers, pharmacists, and individuals of reproductive age who have sex with opposite-sex partners and are thus at risk of unintended pregnancy.

## Access

The U.S. has several forms of emergency contraception available with varying degrees of accessibility. Since August of 2013, Plan B One-Step has been available over-the-counter (OTC) without restriction by age or gender (and all generic forms have been available OTC since 2014), while ella is available by prescription only, and IUDs must be inserted by medical providers [7, 73]. Studies show that, within pharmacies, barriers to access include consumers being able speak to a human on the phone; misinformation from employees (e.g., age restrictions, ID requirements); keeping OTC medication in a locked cabinet or behind the counter; stock-outs; choosing not to carry ella due to low consumer demand; and barriers to pharmacists’ ability to dispense ella without a physician prescription in 43 states [7, 53, 54, 74–76]. Out-of-pocket costs quoted for ella varied widely, ranging from $2.59–$1200.99 with a median cost of $50 [53]. In spite of advances since Plan B One-Step was made available OTC without restriction, systematic barriers still exist that restrict access to emergency contraception [68].

### Gaps in knowledge

Patients often do not realize or appreciate the full range of emergency contraception options that are available [30]. This problem spans across sociodemographic characteristics and neighborhood, although certain groups are less likely to be well-versed and knowledgeable about contraceptive choices [68]. Surveys show there are gaps in knowledge related to age-restrictions, parental consent, confidentiality, what methods are available, efficacy, side effects, and timing [5, 7, 77]. A problem exacerbating lack of knowledge among women at risk of unintended pregnancy is that patients have fewer and more abbreviated opportunities to receive counseling from providers about the most effective forms of emergency contraceptives as well as longer-term contraceptives now that they can acquire EC over-the-counter [10, 78].

Patients are not the only ones with gaps of knowledge around emergency contraceptives; providers are often ill-informed about the options available as well [77]. For providers who do not specialize in reproductive health, there is much less familiarity with ulipristal acetate or IUDs for emergency contraception relative to LNG [6]. In a survey of clinicians in a range of hospital specialties, provider knowledge ranged from 95% knowing of LNG and 62% offering it to only 29% knowing about ulipristal acetate and just 7% providing it [6]. A survey among emergency department providers in Georgia found that only 3% of providers knew the maximum window of efficacy for EC [8].

### Financial barriers

Financial barriers disproportionately impact individuals closer to the federal poverty line, which is also a risk factor for unintended pregnancy [68]. Relative to higher-income neighborhoods, pharmacies in low-income neighborhoods are 50% more likely to have permanent stock-outs of ECPs (odds ratio (OR) 1.5, 95% confidence interval (CI): 1.20–1.94) [74]. Out-of-pocket costs for EC can range from $0–1300 in the U.S. depending on if a woman has insurance or not [59]; the device itself costs $778 [79]. Changes to the Affordable Care Act could potentially impact whether birth control and emergency contraception would be covered in the future [7], but at the time of writing, EC should be covered under the ACA with the caveat that there may be a lack of coverage for employees who work for religious employers or religious nonprofits [80].

In terms of economics, because of the lack of awareness of copper IUDs and ulipristal acetate as the most effective forms of emergency contraception, there is less demand for them [6]. This depresses the supply, as well, feeding into a vicious cycle, as exemplified in Hawaii, where only 2.6% of pharmacies had ulipristal acetate on hand in a phone survey [81]. Providers may also be disincentivized to keep IUDs on hand due to the high upfront costs and concerns about not being fully reimbursed [82].

### Health systems barriers

Several health systems barriers impede a patient’s ability to access emergency contraception. In instances of rape and sexual assault, only 17 states and the District of Columbia (D.C.) have laws in place requiring emergency departments to inform patients about emergency contraception [54]. Thirteen states and D.C. stipulate that EC must be dispensed upon request, although in Pennsylvania, providers may refuse for moral or religious reasons, provided that they transport the patient to the nearest medical facility willing to dispense EC [54]. Religiously affiliated hospitals may restrict access to care relative to secular facilities [83]. In the US, one in ten hospitals beds falls under the purview of the Catholic Directives, a set of guidelines that restricts access to reproductive healthcare, among other services [84].

On the grounds that it impinges on religious freedoms, some pharmacists and pharmacy-owners take issue with stocking and dispensing EC [85–87]. Among those who cite conscientious objection, a reason that is raised is that individuals believe dispensing EC is tantamount to facilitating an abortion, despite evidence to the contrary [87, 88]. In 2015, in *Stormans, Inc. v Wiesman*, the US Ninth Court of Appeals found that pharmacies must fill prescriptions regardless of conscientious objection [87]. A consensus was established by the international human rights community, comprised of the United Nations (UN) Committee on Economic, Social, and Cultural Rights; the UN Committee on the Elimination of Discrimination against Women; the UN Human Rights Committee; the European Court of Human Rights, as well as the American College of Obstetrics and Gynecology and the World Health Organization: “The Primary conscientious duty of healthcare providers is to treat, or to provide benefit and prevent harm to patients; conscientious objection is secondary to this primary duty.” In spite of this, there are 7 states in the U.S. that explicitly allow either pharmacies or pharmacists to refuse EC to a patient and another 4 states that are worded more loosely but permit pharmacies and/or pharmacists to refuse dispensing EC for religious or moral reasons [54].

### Practice barriers

Many providers believe that if they mention advanced provision of emergency contraception, this may lead to risk-taking behaviors among patients, not realizing the utility of it as a backup method in an instance when a long-term method fails [68]. There are no risks of unintended pregnancy that outweigh the benefits achieved by use of emergency contraception according to the Centers for Disease Control and Prevention’s *US Medical Eligibility Criteria for Contraceptive Use, 2016* [40]. This includes women with a history of ectopic pregnancy, cardiovascular or liver disease, thromboembolism, migraines, as well as women who are currently breastfeeding. Neither physical exams nor pregnancy tests need to be performed before administering emergency contraceptive pills [68]. Many providers surveyed are unaware of the most efficacious forms of emergency contraception [6]. Another barrier is lack of training on IUD insertion, which may lead to fewer patients accessing them [68].

## Special populations

While studies show improvements in some areas of access to and knowledge of EC among adolescents, barriers persist among systematically disenfranchised segments of the population.

### Adolescents

Providers have been addressing gaps in access among some groups through counseling those at greatest risk, in particular, adolescents. When adjusting for confounders, providers were 88% more likely to talk about EC to black non-Hispanic patients than white ones (95% CI: 1.05–3.39); 90% more likely to talk to a girl whose mother did not get a high school diploma relative to one whose mother had at least some college education (95% CI: 1.05–3.45), 95% more likely to talk to a girl with two or more sexual partners in the previous 12 months compared to one (95% CI: 1.25–3.05), and 84% more likely to talk to a girl at a Non-Title X public clinic than at a private clinic [10].

Concerns expressed by adolescents related to accessing EC include confidentiality, transportation, and embarrassment [68, 89]. Some providers are conflicted in striking a balance between maintaining confidentiality and adhering to policies and/or legal rulings [89–91]. Using the National Survey of Family Growth, 2011–2015, researchers found differences by race/ethnicity, mother’s level of education, number of partners in the previous 12 months, and source of care in the levels of communication regarding emergency contraception by providers during Pap smears [10]. In a survey of adolescent males, only 42% were aware what emergency contraception was [92]. One study found that among a racially heterogeneous group of adolescents, 44% interpreted the “morning after pill” literally, believing Plan B was only effective for 1 day following an episode of UPI [93].

### Immigrants/non-English speaking patients

When patients are able to access healthcare in their native language or at least one they have attained basic proficiency in, they are more likely to seek timely treatment [68]. Within the US, more than half of all pharmacies offer Spanish language services [68]. Online, the Princeton website not-2-late.com is available in both Spanish and Arabic. Increasing language options improves timeliness of access to emergency contraception [67].

### Sexual assault survivors

Among adult women who are raped or sexually assaulted, it is estimated that 5% become pregnant [94]. Women presenting to a medical provider subsequent to a rape/sexual assault should be offered EC, including a copper IUD, as well as the option of collecting forensic evidence [14]. In instances when a woman chooses to have forensic evidence collected, the provider should conduct a forensic examination prior to insertion of an IUD if that option is selected [14]. In spite of guidelines, only 13 states and DC require emergency departments to dispense EC upon request [54].

### Rural populations

Women in rural areas have higher overall, unintended, and teenage pregnancy rates relative to women in urban and suburban areas in the US [95]. Gaining timely access to emergency contraception is paramount for patients who are not located in major urban centers; Bigbee et al. (2007) found that lack of training and demand were the biggest barriers to stocking EC [96].

### Military/veterans

Both women veterans and active duty servicewomen are at heightened risk of unintended pregnancy relative to the general US population: among active duty women, the rate of unintended pregnancy is 34 per 1000 among officers and 80 per 1000 among enlisted women (OR 2.71, 95% CI 1.99–3.69, *p* < 0.001) [97]. Risk factors for unintended pregnancy include lack of use of contraception [98, 99]; history of military sexual trauma; and inconsistent contraceptive use among women veterans with mental illness with/out a substance use disorder [100].

Women veterans are able to access an advanced provision of emergency contraception through the Veterans Health Administration (VHA) for a $9 copay; there is no copay for those with a service-connected disability or a discharge from Iraq or Afghanistan within the past 5 years [98]. Through TRICARE, active duty servicewomen have coverage for all forms of EC, including LNG, ella, and IUDs. However, like at many civilian locations, the availability may be limited and misconceptions and limited provider availability may reduce access [101]. This can be especially challenging for women stationed overseas as alternative services are not available. Active servicewomen surveyed indicate that it is harder to adhere to a daily oral contraceptive regimen across time zones/long shifts on active duty while deployed [102].

## Continuing areas of research

### Timing of restarting hormonal contraception after Ulipristal acetate

Because ulipristal acetate is an anti-progestin, there is concern that immediate start of a progestin-containing contraceptive could counteract the effect of ulipristal acetate [14]. In one pharmacodynamic study, women took ulipristal acetate and then were given either a placebo or started a desogestrel-containing contraceptive pill the next day [103]. Among women who used the progestin-containing pill, 45% of women ovulated within the next 5 days, compared with 3% in the placebo group [103]. It is of significant clinical concern that hormonal contraceptives could render ulipristal acetate EC ineffective and put the woman at risk for unintended pregnancy, given that previous standard clinical practice was to quick-start an ongoing contraceptive immediately for all women using EC pills who wanted a longer-term method. Desogestrel progestin-only pills are not available in the United States and have a different mechanism of action than other progestin-containing hormones, so it is unclear whether these results apply to all progestin-containing hormones. Until more research is available, it is recommended that women wait 5 days after taking ulipristal acetate to start a hormonal contraceptive method.

### Appropriate dosing based on body weight

Research is ongoing; at this point, evidence suggests that for a person who is overweight or obese, a copper IUD is the best option for emergency contraception. Barring that, ulipristal acetate is more effective than LNG, which is only marginally more effective than no form of EC at all among obese women [13, 14, 30]. Double-dosing of LNG is being investigated at this time [38, 39].

## Conclusion

Given the risks for unintended pregnancy among women of reproductive age who have sex with an opposite-sex partner or partners or are subject to rape or sexual assault, emergency contraception is an important last chance to prevent pregnancy. EC pills should be taken as soon as possible after sex. The most effective EC option is the copper IUD, followed by ulipristal acetate, and lastly LNG. Patients using ulipristal acetate EC should wait 5 days before starting an ongoing progestin-containing hormonal contraceptive subsequent to taking EC. Barriers to accessing EC persist, including lack of awareness among providers and low levels of stocking of EC in many stores and pharmacies. Finally, all EC methods are contraceptives–they prevent pregnancy rather than ending an established pregnancy.

## Abbreviations

ACA: Affordable Care Act
BMI: Body mass index
CI: Confidence interval
DC: District of Columbia
EC: Emergency contraception
ECP: Emergency contraception pill
FDA: Food and Drug Administration
Hx: History
IUD: Intrauterine device
kg/m: Kilograms/meter
LNG: Levonorgestrel
mcg: Microgram
mg: Milligrams
N/A: Not applicable
OR: Odds ratio
OTC: Over-the-counter
UN: United Nations
UPA: Ulipristal acetate
UPI: Unprotected intercourse
VHA: Veterans Health Administration
WHO: World Health Organization
